# Supplementary material for: Serial fermentation in milk generates functionally diverse community lineages with different degrees of structure stabilization
Source: mSystems. 2024 Jul 23;9(8):e00445-24. doi: 10.1128/msystems.00445-24 (PMC11334471; doi:10.1128/msystems.00445-24)
Supplement: Supplemental figures — Figures S1 to S6. [file msystems.00445-24-s0001.docx]

**Supplementary figures** for the article “Serial fermentation in milk generates functionally diverse community lineages with different degrees of structure stabilization”

**
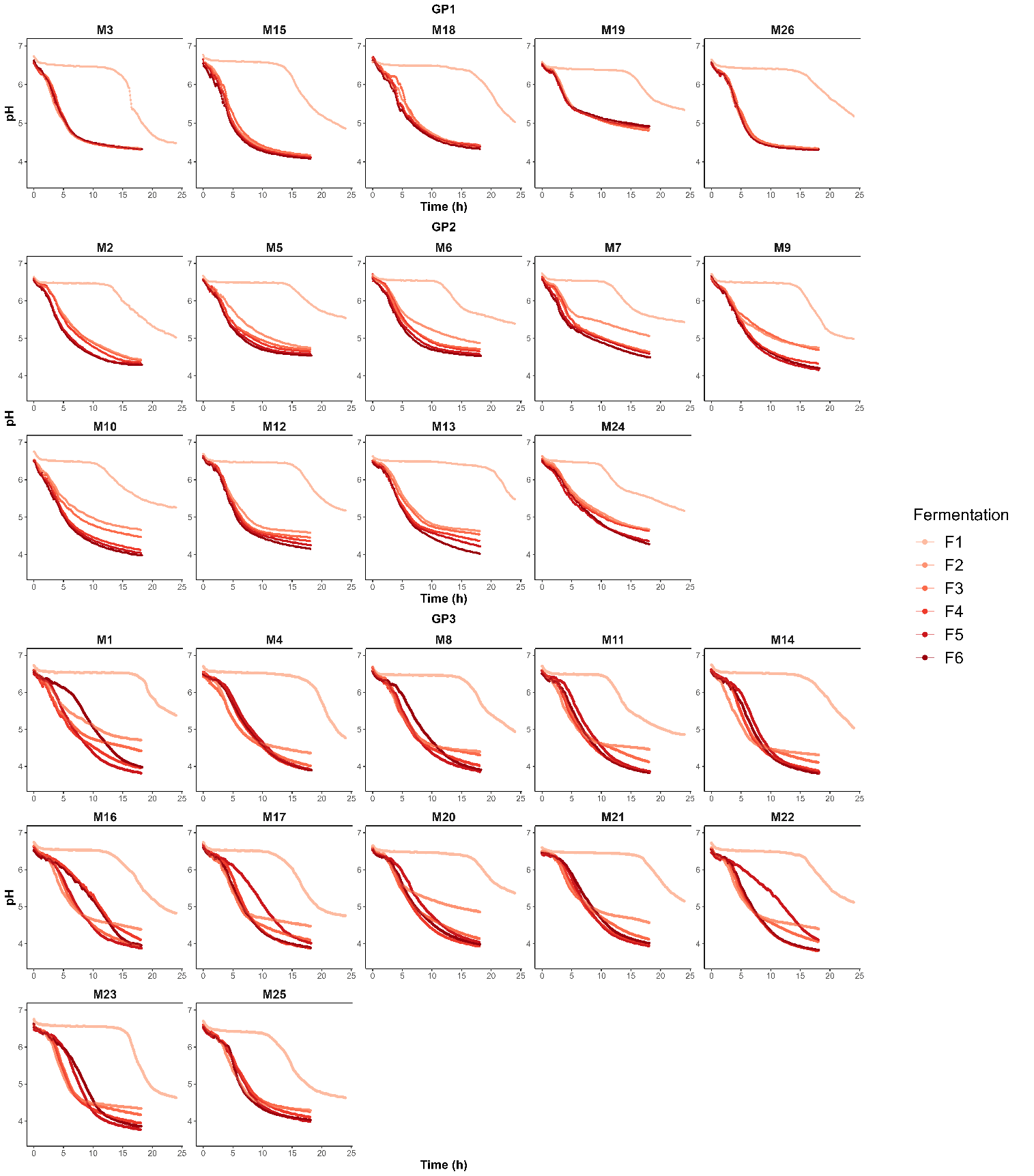
**

**Figure S1.** **Acidification kinetics of all raw milk samples during serial fermentation.** The colours indicate the fermentation step, from light pink (F1) to dark red (F6).


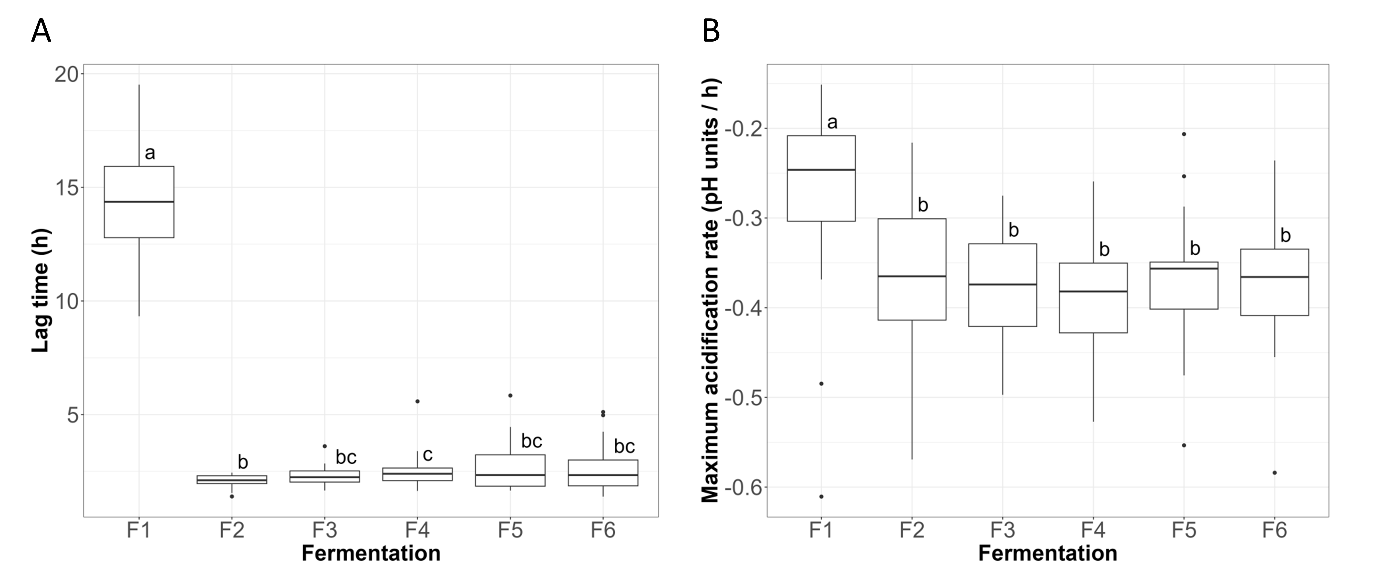


**Figure S2. Acidification parameters according to the fermentation step (F1-F6) for all samples.** (A) Lag time and (B) MAR. Different superscript letters indicate a significant difference (*p* < 0.05; pairwise comparisons using the Wilcoxon rank sum exact test with the Bonferroni adjustment method for the *p* value).


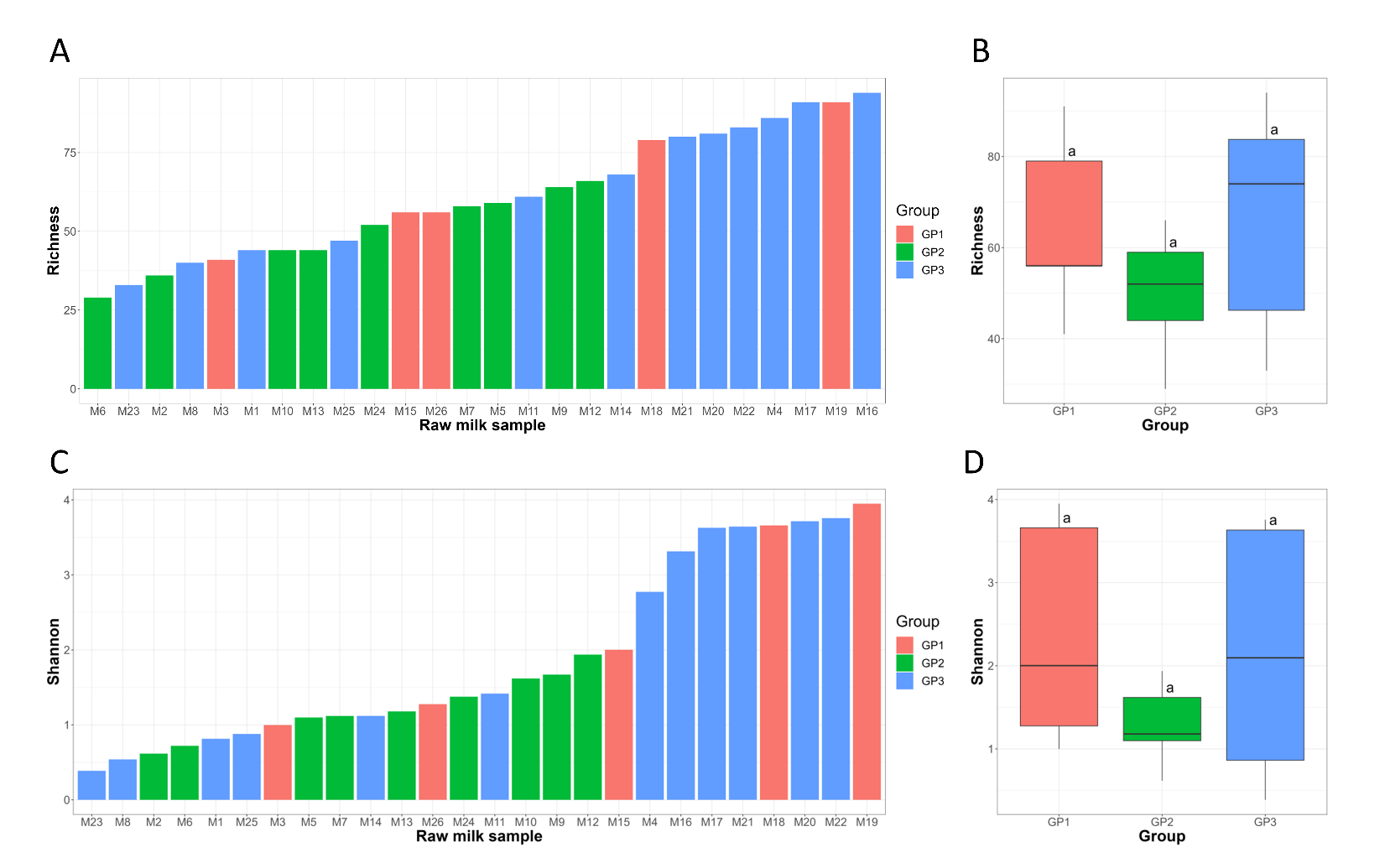


**Figure S3. Alpha diversity (richness and Shannon index) of the raw milk samples.** (A, C) According to the milk sample and (B, D) according to the acidification group. The same superscript letters indicate no significant difference (*p* > 0.05; one-way ANOVA).


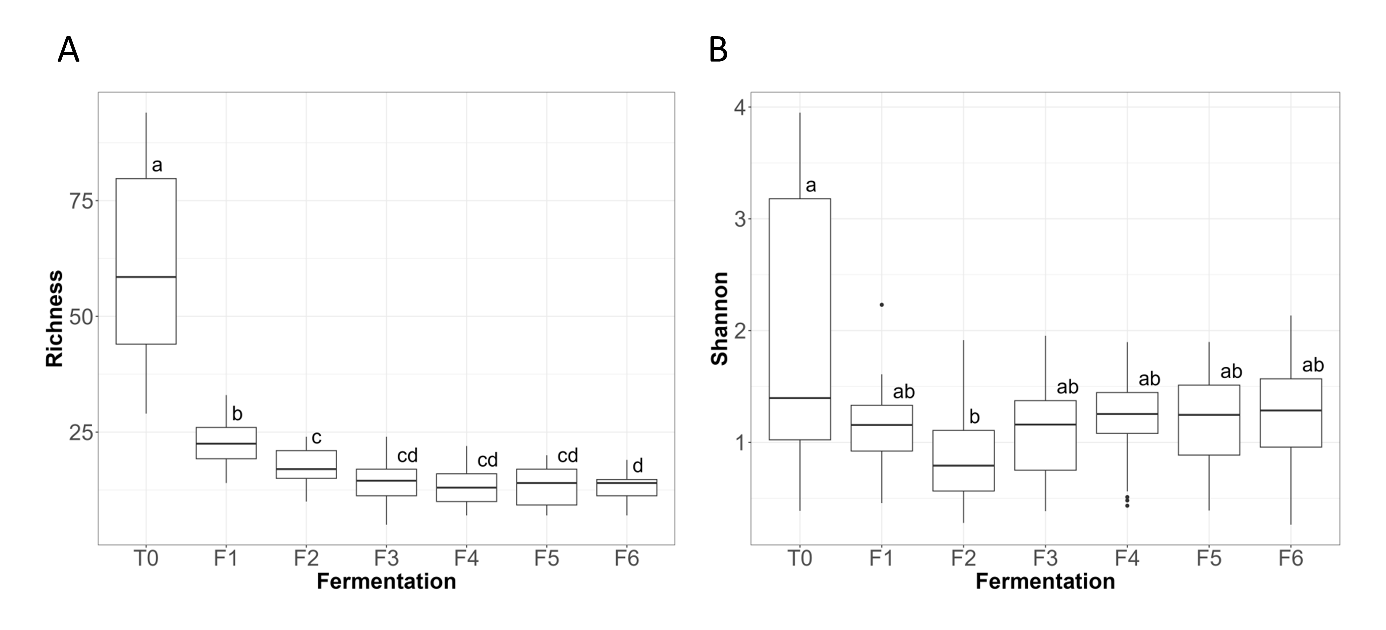


**Figure S4. Alpha diversity of all communities according to the fermentation step (T0-F6).** (A) Richness, (B) Shannon index. Different superscript letters indicate a significant difference (*p* < 0.05; Pairwise comparisons using the Wilcoxon rank sum exact test with the Bonferroni adjustment method for the *p* value).


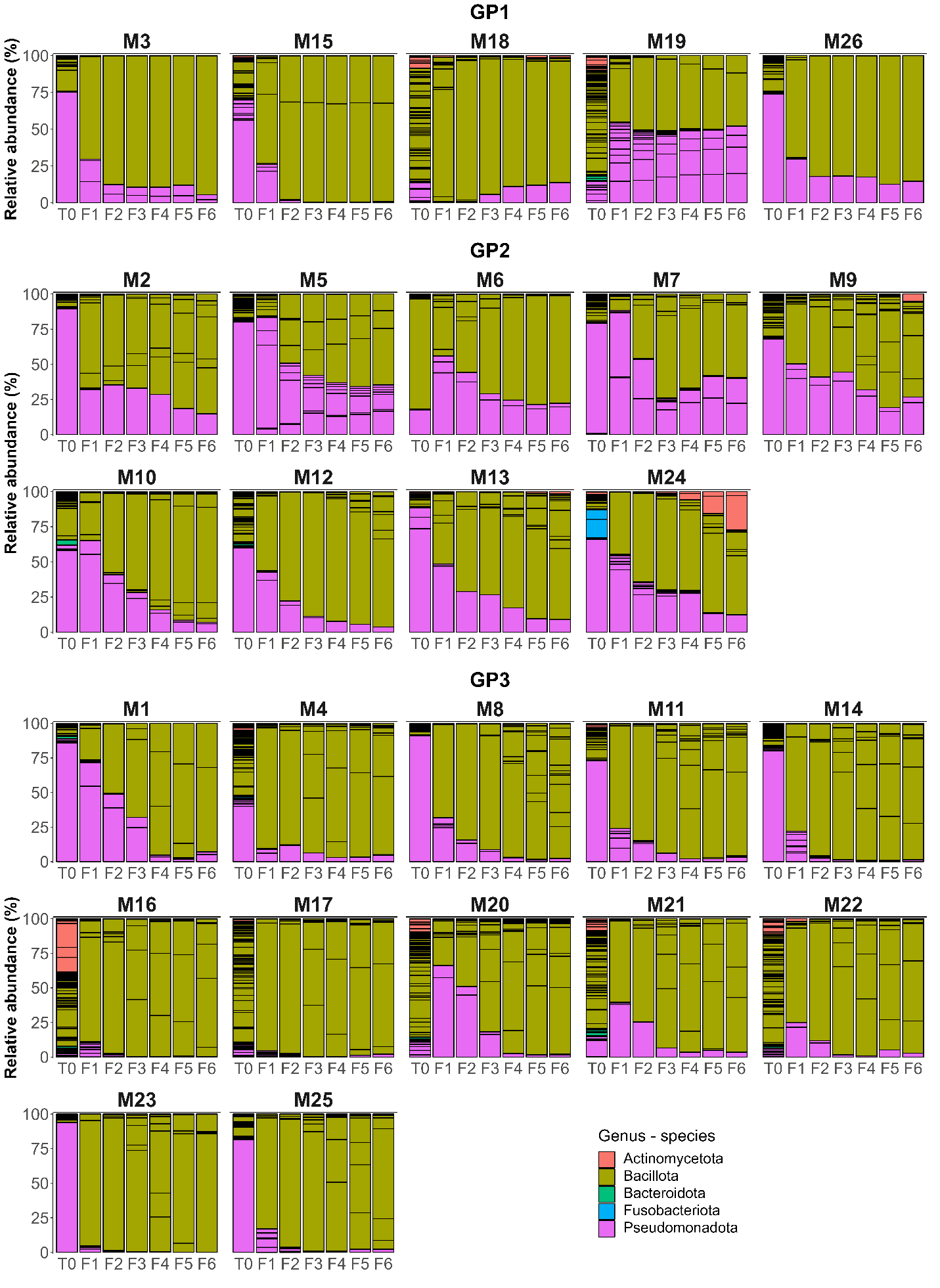


**Figure S5.** **Bacterial community composition at the phylum level for each raw milk sample and fermentation step.** T0: raw milk samples, F1 to F6: fermented milk samples.


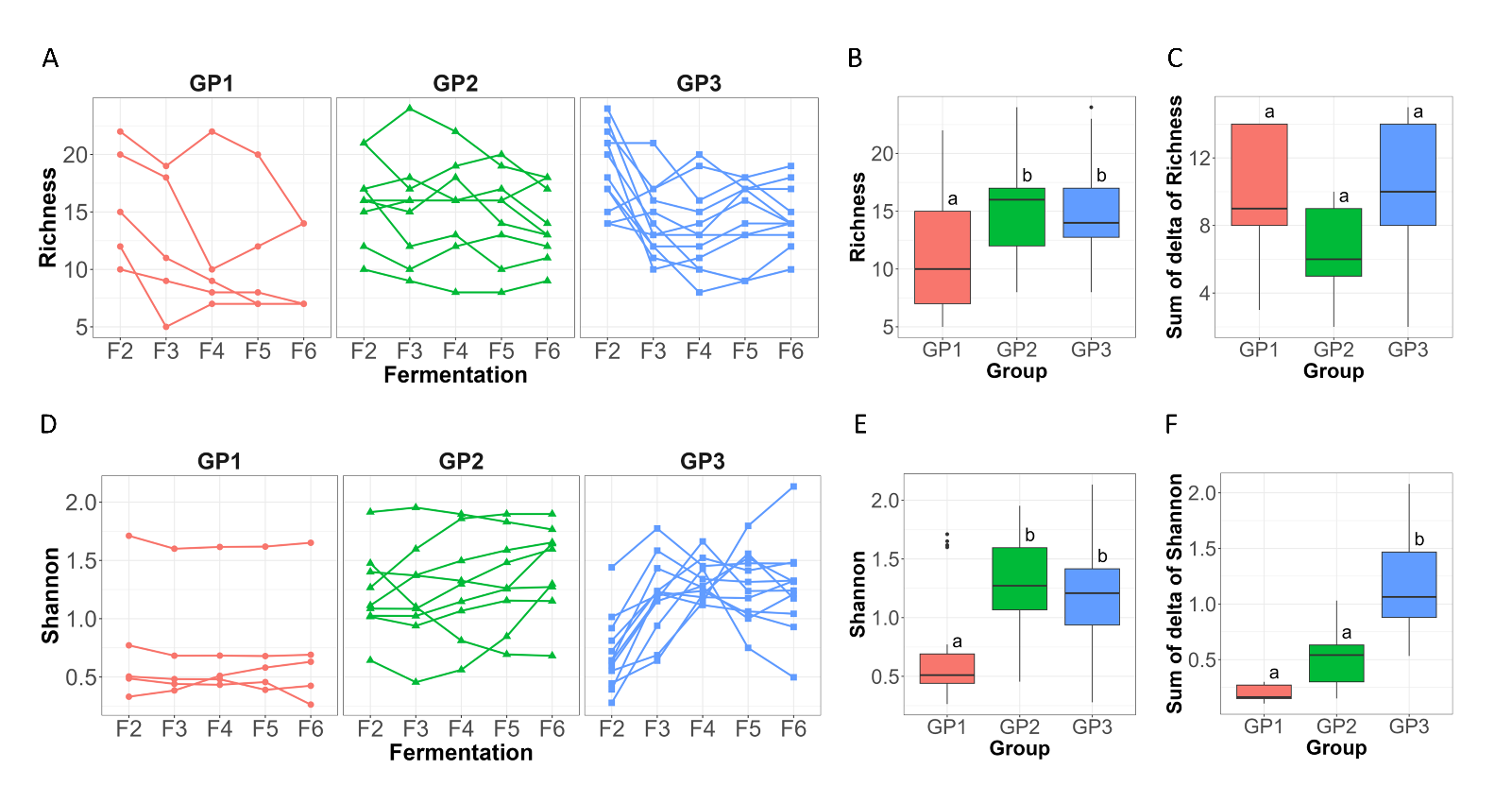


**Figure S6. Alpha diversity analysis.** (A, B) Richness for each lineage and overall (F2-F6), and (C) sum of richness variations between adjacent steps, for each group GP1, GP2 and GP3. (D, E) Shannon indices for each lineage and overall (F2-F6), and (F) sum of Shannon indices for each group. Different superscript letters indicate a significant difference (*p* < 0.05; Pairwise comparisons using the Wilcoxon rank sum exact test with the Bonferroni adjustment method for the *p* value).
